# The Association between Academic Performance and Entry-to-Practice Milestones within a Co-Operative Education PharmD Program

**DOI:** 10.3390/pharmacy12030090

**Published:** 2024-06-11

**Authors:** Ali Syed, Yuying Huang, Joslin Goh, Sarah Moroz, John Pugsley, Nancy M. Waite, Sherilyn K. D. Houle

**Affiliations:** 1 School of Pharmacy, University of Waterloo, 10 Victoria St S A, Kitchener, ON N2G 1C5, Canadanancy.waite@uwaterloo.ca (N.M.W.); 2 Department of Statistics and Actuarial Science, University of Waterloo, 200 University Avenue West, Waterloo, ON N2L 3G1, Canadajoslin.goh@uwaterloo.ca (J.G.); 3 Office of Institutional Planning & Analysis, York University, 4700 Keele St, Toronto, ON M3J 1P3, Canada; semoroz@yorku.ca; 4 Pharmacy Examining Board of Canada, 59 Hayden St Suite 200, Toronto, ON M4Y 0E7, Canada; jpugsley@pebc.ca

**Keywords:** pharmacy, education, experiential education, curriculum, practice readiness, pedagogy, assessment

## Abstract

Research on associations between student performance in pharmacy programs and entry-to-practice milestones has been limited in Canada and in programs using a co-operative (co-op) education model. Co-op exposes students to a variety of opportunities both within direct patient care roles and in non-traditional roles for pharmacists, such as policy, advocacy, insurance, research, and the pharmaceutical industry. The purpose of this research is to analyze associations between student grades and evaluations achieved in the University of Waterloo (UW) Doctor of Pharmacy (PharmD) co-op program and success rates on entry-to-practice milestones, including the Pharmacy Examining Board of Canada (PEBC) Pharmacist Qualifying Examination and performance on final-year clinical rotations. Grades and evaluations from courses, co-op work terms, clinical rotations, and PEBC exam data from three graduating cohorts were obtained. A multiple regression analysis was performed to explore associations between student evaluations and PEBC Pharmacist Qualifying Examination and clinical rotation performance. Holding all other variables constant, grades in anatomy/physiology were negatively correlated with scores on the PEBC Pharmacist Qualifying Examination, while grades in one of the professional practice courses showed a positive relationship with the same examination. Students with higher grades in a problem-based learning capstone therapeutics course, in their first co-op work term, and in the direct patient care co-op work term tended to score higher on clinical rotations. Co-op performance was not significant in predicting PEBC performance. However, complimentary descriptive analysis underscored that students with a co-op rating of good or below were more likely to fail courses, midpoint evaluations, Objective Structured Clinical Examinations (OSCEs), and PEBC measures. Multiple predictors of performance on final-year clinical rotations and the PEBC Pharmacist Qualifying Examination were identified. This predictive model may be utilized to identify students at risk of underperforming and to facilitate early intervention and remediation programs, while also informing curricular revision.

## 1. Introduction

Health professional degree programs face challenges with adequately preparing new graduates for practice in a dynamic healthcare system [1,2]. Misalignment between competencies assessed within health professional curricula and those applied within healthcare systems may compromise the student-to-practitioner transition and contribute to new graduates’ feelings of professional inadequacy [2]. In Canada, the pharmacy education system has evolved in response to emerging and expanded roles for pharmacists, which require a greater focus on clinical interventions and interprofessional collaboration to optimize patient care [3].

Adequately preparing pharmacy graduates for practice involves continuous curricular evaluation, with experiential placements crucial for developing discipline-specific skills [4]. Addressing the challenge of aligning pharmacy education with the dynamic healthcare landscape may involve incorporating work-integrated learning (WIL), such as co-operative (co-op) education [5]. Co-op integrates didactic education and practical work experience, promoting continuous learning and enhancing job readiness by identifying essential competencies for practice in response to current and future healthcare needs [6,7]. However, with only two co-op pharmacy programs in North America, there is a research gap regarding the association between academic performance within a co-op program and subsequent performance in entry-to-practice assessments.

To optimize curriculum and identify students who would benefit from additional support, it is crucial to explore potential predictors of readiness, like student grades, milestone assessments, and experiential placements. These predictors, when correlated with established surrogates of readiness, such as certification/licensure exam pass rates, help appraise components of pharmacy education. Studies have identified predictors for performance on pharmacist licensing exams, such as pre-admission grade point average (GPA), performance on the Pharmacy College Admission Test (PCAT), and pharmacy program GPA [8,9,10]. However, the concept of readiness for entry-to-practice lacks a consistent definition and varies across practice settings. Generally, it refers to possessing the knowledge, skills, and judgment required for a specific role [11]. Recognizing the evolving nature of pharmacy education and the unique characteristics of our co-op program at the University of Waterloo (UW), a focused analysis is necessary to understand the specific factors influencing readiness for entry-to-practice among our graduates. 

In the United States, pharmacists must pass the North American Pharmacist Licensure Examination (NAPLEX) to obtain licensure, while in Canada, the standard exam is the Pharmacy Examining Board of Canada (PEBC) Pharmacist Qualifying Examination, which includes a Part I Multiple-Choice Question (MCQ) and a Part II Objective Structured Clinical Examination (OSCE) [12,13]. In the United Kingdom, pharmacists take the General Pharmaceutical Council (GPhC) Registration Assessment [14]. Similarly, in Australia, pharmacists must take the Australian Written Intern Examination to secure their licensure [15]. Shah et al. emphasized the importance of identifying reliable predictors of NAPLEX outcomes and students at risk of poor performance [12]. This is crucial not only for ensuring the competence of future pharmacists, but also for informing educational strategies and interventions aimed at supporting students’ success. Similar concerns likely exist within the Canadian context, where understanding factors influencing performance on the PEBC Pharmacist Qualifying Examination is equally essential for maintaining high standards in pharmacy practice [8].

### Program Overview

The UW PharmD program, Canada’s only co-operative (co-op) undergraduate pharmacy program and one of two in North America, begins with foundational sciences and an introduction to professional practice in year one [16,17]. In years two to four, students delve into Integrated Patient Focused Care (IPFC), a series of nine courses covering therapeutic areas by system, including pathophysiology, pharmacology, pharmacokinetics, critical appraisal, and clinical problem-solving [17]. From year two, students also undergo annual OSCE exams, and a midpoint exam is completed in year two to identify at-risk students and offer remediation [17].

**Figure 1:**
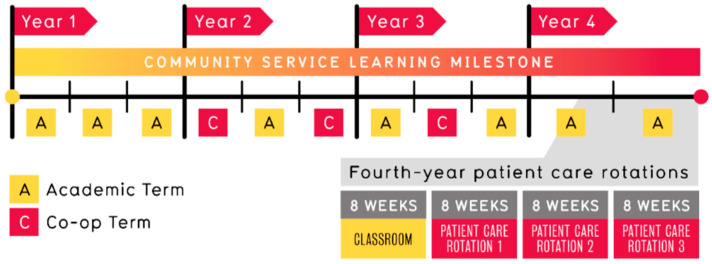
University of Waterloo PharmD program curricular sequence.

Experiential education consists of a Community Service Learning (CSL) milestone, co-op work terms, and final-year clinical rotations:

1. CSL is a mandatory non-course degree requirement and milestone in the PharmD program [17]. CSL consists of three phases, preparation, action, and application, aimed at engaging PharmD students in service activities, reflecting on personal and professional goals, and contributing to community health [17].
2. The co-op program mandates three employer-paid 16-week full-time work terms, with the initial two work terms occurring in the second year and the final one in the third year [7]. Students can experience various work settings, including primary care, institutional pharmacy, industry, government, and research [7]. They can secure placements through competitive applications and job interviews with school-recruited positions or self-arrangement [7]. Student responsibilities align with the employer’s job description, with terms and passing requirements detailed in Table 1 [7].
3. During clinical rotations, students assess patients, address drug-related needs, devise and execute care plans, and co-operate with interprofessional teams to enhance patient outcomes [18]. These unpaid courses require students to complete all three rotation placements within a lottery-assigned region in Ontario [18]. Course numbers correspond to the care setting, including institutional, primary care, and elective rotations [18].
4. Additional information on the curriculum is provided in (Figure 1) and in Supplementary Figure S1. A complete list of abbreviations can be found in Supplementary Table S1.

The research to date examining relationships between pharmacy student performance in academic programs and entry-to-practice assessments is limited and does not encompass programs with unique curricular features, like co-op [8,9,10,19]. Notably, there is growing interest in the co-op model as a robust WIL model. This paper aims to fill this knowledge gap by investigating whether course grades and evaluations, including co-op work terms and direct patient care rotation courses, correlate with performance on entry-to-practice measures in a Canadian co-op pharmacy program.

## 2. Materials and Methods

Two predictive validity studies were conducted using a retrospective multiple-cohort design: the first explored the relationship between didactic and experiential scores and performance on the PEBC Pharmacist Qualifying Examination Parts I and II ((MCQ and OSCE), while the second explored the relationship between didactic and experiential scores and performance on clinical rotations. Ethics approval for this study was received from the Research Ethics Board at the University of Waterloo (ORE# 43127).

### 2.1. Data Collection

Academic performance data were obtained from the Office of the Registrar and School of Pharmacy for all students (*n* = 343) that graduated from the UW PharmD program between 2017 and 2019 to capture data within a consistent curricular window. Specifically, the UW entry-to-practice PharmD degree was first offered to the graduating class of 2015, representing a transition from a previous Bachelor of Science in Pharmacy degree. Data from the 2015 and 2016 cohorts were excluded since modifications were being made to the PharmD curriculum before reaching a consistent structure for the 2017 graduating cohort. Data from the graduating classes of 2020 onwards were also excluded due to the impact of the COVID-19 pandemic on the PharmD curriculum. Data utilized including assessment(s) and minimum requirements are outlined in Table 1 [7,17,18]. CSL data were not analyzed as it is a non-course, credit/no-credit milestone, focusing on experiences that would vary significantly between students, which would be challenging to standardize for incorporation into a regression model. Performances on the MCQ and OSCE components of the PEBC Qualifying Examination were provided by PEBC under a blinded, anonymized, data-sharing agreement.

Students’ identifiable information (e.g., name and student number) was de-identified by a co-investigator data steward to maintain anonymity before being released to the rest of the research team for analysis.

### 2.2. Data Exploration

Correlation coefficients among numerical predictor variables were explored prior to the development of a regression model to avoid a compromised interpretation of the regression. In general, highly correlated predictor variables can be explained by similarities between course type, theme, and/or structure. Term average scores as one of the predictor variables were found to be highly correlated with one or more courses and were excluded from the regression model. High-correlation coefficients (denoted by r) among the remaining predictor variables are listed in Supplementary Table S2, in which correlations greater than 0.7 were considered highly correlated.

### 2.3. Data Analyses

Two analyses were separately performed using multiple linear regressions with backward stepwise selection. Backward selection starts the regression models with all predictor variables and drops a predictor variable from the regression model one at a time until regression models reach their best Akaike information criterion (AIC) performance [20]. The backward selection approach assisted in the selection of relevant predictor variables with the goal of developing a parsimonious and efficient model. To prevent any variables of particular interest from being eliminated by backward selection, the research team identified critical courses/inputs and ensured their inclusion in the model. The following were selected based on a consideration of those that had demonstrated statistically significant correlations with certain entry-to-practice milestones in the published literature, and those that are compulsory components of the curriculum with direct applicability to the provision of patient care [8,9,10,12,13,17,18,19,21,22,23,24,25,26,27]:

- Integrated Patient-Focused Care series courses: PHARM 220, 221, 222, 223, 320, 321, 323, 324, and 422;
- Professional Practice series courses: PHARM 129, 130, 228, 229, 329, and 330;
- Pharmacokinetics: PHARM 224;
- Co-op work-term scores;
- PharmD rotation scores;
- Midpoint assessment MCQ scores;
- Year 1, 2, 3, and 4 OSCE scores.

### 2.4. Analysis 1—The Relationship between Student Course and Experiential Education Performance with PEBC Pharmacist Qualifying Examination Performance (Part I MCQ and Part II OSCE)

Four multiple linear regressions were performed. As there is a high degree of similarity between OPPCAT scores and the overall rotation course scores (i.e., OPPCAT accounts for 85% of the associated rotation course score), we used OPPCAT and rotation course scores as predictor variables separately in the analysis to avoid issues with multicollinearity. Except for the OPPCAT or the rotation course, all four regressions included the same didactic scores and co-op work-term scores for the backward selection, with the following variations related to rotation performance and dependent variables:

- Variation 1: OPPCAT only as predictor variable for rotation performance with PEBC Pharmacist Qualifying Examination Part I (MCQ) as the dependent variable;
- Variation 2: OPPCAT only as predictor variable for rotation performance with PEBC Pharmacist Qualifying Examination Part II (OSCE) as the dependent variable;
- Variation 3: Rotation course overall as predictor variable for rotation performance with PEBC Pharmacist Qualifying Examination Part I (MCQ) as the dependent variable;
- Variation 4: Rotation course overall as predictor variable for rotation performance with PEBC Pharmacist Qualifying Examination Part II (OSCE) as the dependent variable.

### 2.5. Analysis 2—The Relationship between Didactic Courses and Co-Op Performance with Rotation Performance

Six multiple linear regressions were performed. All six regressions included the same didactic scores and co-op work-term scores as predictor variables for the backward selection, with variations in dependent variables related to rotations performance consistent with the approach taken previously:

- Regression 1: OPPCAT only from the first completed rotation as the dependent variable;
- Regression 2: OPPCAT only from the second completed rotation as the dependent variable;
- Regression 3: OPPCAT only from the third completed rotation as the dependent variable;
- Regression 4: PHARM 430 (Primary Care) rotation overall score as the dependent variable;
- Regression 5: PHARM 440 (Institutional) rotation overall score as the dependent variable;
- Regression 6: PHARM 450 (Elective) rotation overall score as the dependent variable.

### 2.6. Model Diagnostics

All relevant model diagnoses (i.e., normal QQ plot and residual diagnostic plot) were conducted to examine the model fit, which found that no obvious linear model assumptions were violated across all analyses. The variance inflation factor was also computed for each analysis, which suggests that the statistical models do not have multicollinearity issues.

### 2.7. Complimentary Descriptive Analysis

We compared the rates of course failure and near failure (defined as 60–70% for didactic courses, 60–65% for annual OSCE, and 70–75% for rotations courses) between students that received a marginal, satisfactory, and/or good score across at least one co-op work term and those scoring very good and above across all work terms.

## 3. Results

In this section, we will present the results obtained from the regression models. Both Table 2 and Table 3 only show the variables selected by the backward selection with a 0.05 significance level. Coefficients are estimated with the maximum likelihood estimation method and describe the relationship between the predictor and dependent variables.

### 3.1. Analysis 1—The Relationship between Student Course and Experiential Education Performance with PEBC Exam Performance (MCQ and OSCE)

Excluding the missing data, analysis 1 includes 301 students, with 50 candidate predictor variables and two response variables (PEBC MCQ and OSCE scores). Only those variables selected by backward selection with significant coefficients in the regression models are reported in Table 2.

### 3.2. Analysis 2—The Relationship between Didactic Courses and Co-Op Performance with Rotation Performance

Excluding the missing data, analysis 2 includes 308 students with 47 candidate predictor variables and three response variables (OPPCAT 1, 2, and 3 scores). Only those variables selected by backward selection with significant coefficients in the regression models are reported in Table 3.

### 3.3. Complimentary Descriptive Analysis

Of the 343 students included in this analysis, 85% received overall CSPE ratings above the good level (i.e., very good, excellent, and/or outstanding) across all three co-op work terms, seven students (2%) received at least one overall CSPE rating below the good level (i.e., marginal and/or satisfactory), and 44 students (13%) received at least one overall CSPE rating of good. Inferential analysis will not produce meaningful results due to the highly imbalanced grade distribution. As seen in the summarized findings in Table 4, each cell refers to a subgroup of students. Given the small subgroup of students, there is an overlap between students in each category.

## 4. Discussion

This is the first study to assess performance on the Canadian PEBC Pharmacist Qualifying Examination and final-year clinical rotations within a pharmacy program using a co-operative education model. Positive and negative coefficients were identified from linear models based on pharmacy school performance as well as PEBC-Part I (MCQ) and Part II (OSCE) outcomes. Notably, co-op experiences showed no significant association with PEBC Pharmacist Qualifying Examination outcomes. Furthermore, the conversion of PharmD rotation OPPCAT scores to overall PharmD rotation grades yielded comparable coefficients with the PEBC Pharmacist Qualifying Examination, emphasizing the predictive value of overall rotation course performance. Descriptive statistics revealed a relationship between subpar co-op performance and multiple program milestones, underscoring the importance of CSPE ratings as predictive indicators for program success.

This study employs multiple linear regression analysis to identify predictors of performance, providing a comprehensive assessment of practice readiness. Our study identifies key predictors of PharmD rotation and PEBC performance during a consistent curricular window, offering an opportunity for early intervention with at-risk students. As the PEBC Pharmacist Qualifying Examination is nationally administered, this model may be replicated by other Canadian schools of pharmacy to identify their own predictors of success on practice readiness milestones [13]. This model uniquely includes co-op performance variables as well as both formative and summative measures, enhancing its utility and relevance. This model is also the first to include a midpoint evaluation, as other studies have only explored the Pharmacy Curriculum Outcomes Assessment (PCOA), which is analogous to a midpoint assessment across pharmacy schools in the United States [24,26]. Additionally, it suggests potential curriculum revisions to optimize student success, such as re-evaluating course weight, structure, and assessments based on identified associations, particularly courses with inverse relationships to performance in clinical rotations and the PEBC Pharmacist Qualifying Examination [27].

We identified several positive regression coefficients between pharmacy school performance and PEBC QE Part I (MCQ) outcomes. Noteworthy associations included scores in professional practice (PP) 4, IPFC 8, year 4 OSCE, midpoint MCQ, and clinical rotation 3 OPPCAT. Courses such as PP 4, following a hybrid model of didactic lecturing and laboratory time involving practice-based assessments, and IPFC 8, involving didactic lecturing in a large classroom environment alongside frequent, rigorous assessments, demonstrated positive associations with PEBC QE I (MCQ) outcomes. These findings suggest that courses taken later in the program, focusing on pharmacy practice and therapeutic content, as well as the assessment of patient care competencies, may contribute to the observed coefficients.

Negative coefficients between performance in pharmacy-school and PEBC QE I (MCQ) outcomes were also identified. Notable negatively correlated variables included scores in Anatomy and Physiology 2 and PP 6. Anatomy and Physiology 2 is a didactic, instructor-led course alongside a wet lab, prior to any patient care experiences, and success in this course is largely dependent on rote memorization. Like PP4, PP6 follows a hybrid model of didactic lecturing and laboratory time, while addressing additional topics, such as job acquisition, negotiations, drug shortages, ethics, and professionalism, expanded scope, and documentation strategies. These discrepancies in content relevance and assessment methodologies may explain the observed negative coefficients.

Similarly, positive coefficients were found between pharmacy-school performance and PEBC QEII (OSCE) outcomes, particularly with courses such as PP 3, PP 4, and the symposium (where students present seminars and demonstrate their skills in evaluating literature and synthesizing information in written and oral formats) emphasizing pharmacy practice experiences, patient care, communication, and engagement. Conversely, courses such as Anatomy and Physiology 2 and Fundamentals of Business Administration and Management exhibited negative associations with PEBC OSCE outcomes, possibly due to the stark differences in content relevance and competency assessment structures offered in these courses versus a practical clinical assessment.

Both parts of the PEBC Qualifying Examination were positively related with PP 4 scores and negatively correlated with Anatomy and Physiology 2. Interestingly, no association was observed with co-op evaluations, suggesting divergent competencies assessed by co-op, (e.g., job performance, interest in work, quality of work, teamwork, resourcefulness, entrepreneurial orientation, response to supervision, etc.) and the PEBC Qualifying Examination, which focuses on entry-to-practice competencies of pharmacists.

Positive relationships between performance in pharmacy-school and final-year PharmD clinical rotation OPPCAT outcomes were identified, notably with scores in IPFC 9 and co-op direct patient care. This may be since IPFC 9 is the final therapeutics course in the program and applies a problem-based learning approach in a self-directed format, which may draw similarities with clinical practice. The positive coefficients between co-op direct patient care scores and rotation OPPCAT 3 scores may result from similar competencies assessed in both, with OPPCAT 3 reflecting improved clinical performance as the final PharmD rotation. Conversely, negative relationships were found with scores in IPFC 3. Despite structural similarities across most IPFC courses, the reasons for this negative relationship are uncertain and may involve factors such as course content or teaching and assessment styles, which will be examined in future work.

Descriptive statistics reveal a relationship between subpar co-op performance and multiple milestones in the UW PharmD curriculum. Students with below-good CSPE ratings were 3.5-times more likely to fail courses and 1.3-times more likely to experience near failures compared to those with good or above ratings. Additionally, they showed higher probabilities of failing the OPPCAT (6.3-fold), PEBC Part I (MCQ) (6-fold), PEBC Part II (OSCE) (4.7-fold), midpoint (2-fold), and annual OSCE (1.4-fold). Similarly, students with “good” CSPE ratings were more likely to experience failures or near failures across various measures, suggesting an association between CSPE ratings and program success. These findings underscore the importance of early identification and intervention for students exhibiting lower CSPE ratings, potentially indicating a need for targeted support mechanisms to improve overall program outcomes. Furthermore, the consistently higher probabilities of failure across multiple assessments for students with below-good CSPE ratings highlight the significance of addressing underlying issues in co-op performance as a potential predictor of broader academic challenges.

Our study supports the findings of previous studies exploring predictors of performance in pharmacy school and on pharmacist licensing exams, while introducing additional predictors [10,12,24,25,26]. In a study by Call et al., students who received a failing grade on one or more advanced pharmacy practice experiences (APPEs) were compared to other students that passed; however, this was performed using a retrospective approach specific to one cohort and did not involve the use of a predictive model [19]. In close alignment with our study, Shah et al. and Cor et al. developed a predictive model to identify students at risk of poor performance on the NAPLEX and isolated pre-admission and pharmacy program predictors [12,21]. Shah et al. found their predictive model to be a practical tool in which four of the five predictors could be generalizable to other schools of pharmacy [12]. Similarly, Cor et al. found that biology-based prerequisites had a consistent relationship with academic performance, suggesting a need for reconsideration of admissions GPA criteria and the inclusion of non-traditional predictors to better assess student success [21].

Studies by McCall et al., Chisholm-Burns et al., Allen et al., Garavalia et al., and Elder et al. also identified significant pharmacy program predictors of NAPLEX performance [10,22,23,24,25]. McCall et al. identified the composite PCAT score as the primary predictor for NAPLEX outcomes, yet the combined predictive power of PCAT, Critical Thinking Skills Test scores, prepharmacy GPA, and age was relatively low, emphasizing the need for a comprehensive review of each candidate’s application [22]. Chisholm-Burns et al. stressed the critical role of first-year pharmacy GPA in predicting on-time graduation and NAPLEX success, underscoring the importance for close monitoring of student performance in the initial year and proactive support for those facing academic challenges [10]. Allen et al. identified no unsatisfactory grades in the prepharmacy program and a high cumulative GPA in the PharmD program as significant predictors of success on the NAPLEX, emphasizing the importance of academic performance throughout the program [23]. Garavalia et al. emphasized the correlation between GPA, PCOA scores, and NAPLEX performance, stressing the importance of validating PCOA scores for academic decision making [24]. Elder et al. found that performance on specific pharmacy clinical skills lab assessments predicted total NAPLEX scores, indicating the need for early intervention for struggling students [25]. However, these studies focus on the NAPLEX, which differs in structure and competencies compared to the PEBC. Furthermore, these studies analyzed one measure of practice readiness, as opposed to examining multiple measures of practice readiness and teasing out mutual predictors between them.

A Canadian study conducted by Cameron and colleagues explored predictors of performance on the PEBC Qualifying Examination among pharmacy students at the University of Toronto [8]. This study found that the Multiple Mini Interview (MMI), measuring non-academic attributes, was the sole admissions tool with significant predictive validity for performance in the PEBC-OSCE and institutional/ambulatory rotation, emphasizing its importance in health professions student selection [8]. However, this program does not offer co-op work terms and therefore may not be generalizable to our program.

The findings offer valuable insights for pharmacy education institutions, providing a foundation for early intervention strategies to support students at risk of underperforming. Additionally, the predictive model developed in this study could benefit other Canadian schools of pharmacy in identifying predictors of success on practice readiness milestones.

Future research could explore additional factors impacting pharmacy licensure exam and clinical rotation performance, such as personality profiles and resilience. While this study uses the PEBC exam and PharmD rotations as proxies for practice readiness, further research should consider other factors, such as cognitive, clinical, and professional capabilities, as well as self-efficacy that may influence both academic performance and practice readiness. Without a widely accepted definition for practice readiness, employer and preceptor perspectives on student preparedness should also be explored. Finally, future research could adopt an extended longitudinal approach that follows students through their initial years of practice to understand their transition from student to new graduate to more established healthcare professional.

### Limitations

This study does not establish a direct cause-and-effect relationship with the identified predictors. It is important to note that our data collection was limited to student academic performance and cohort year; therefore, we are unable to comment on other individual characteristics, such as age, sex, gender, completion of another degree before entering pharmacy school, and GPA upon admission. This study was a single-institution study with a unique co-op program. In addition, more recent cohorts were affected by the COVID-19 pandemic, which may limit generalizability toward future cohorts. As PharmD students may not satisfy the communication, distribution, and direct patient care requirements of the co-op program in a fixed order and may satisfy them more than once on a given co-op term, it was necessary to average these scores across the three terms. Averaging may underrepresent the information contained in the co-op data. Finally, it is important to note that the PEBC Pharmacist Qualifying Examination is an assessment of minimal competency; therefore, assessing additional measures of practice readiness that go beyond minimal competency or that cannot be assessed by the examination format is encouraged for future studies. 

## 5. Conclusions

Multiple significant predictors of performance on PharmD clinical rotations and the PEBC exam were identified. Subpar performance on co-op correlated with poor performance on a number of curricular milestones. These curricular performance predictors can be used to identify students at risk of underperforming, to facilitate early intervention and remediation programs, as well as inform curricular revision. Future studies should evaluate and propose a standardized definition for practice readiness for pharmacists and explore qualitative perspectives of employers and preceptors on student and new graduate preparedness for practice. Educators are encouraged to pay particular attention to students’ performance in professional practice and therapeutics courses, as a poor performance in these may indicate a need for additional support to ensure success in final-year rotations and licensure exams.

## Figures and Tables

**Table 1:** Data utilized in multiple linear regression analyses [7,13,17,18].

Evaluation	Assessment(s) and Minimum Performance Required to Pass
Didactic courses *	Final grade of 60% or higher
Co-op work terms *	PharmD Inventory of Skills Evaluation (Supplementary Figure S2): Overall rating of “good” or higher for each of: ○ Communication (all work terms) ○ Drug distribution (work term 1 or 2) ○ Direct patient care (work term 2 or 3) Overall rating of “meets expectations” or higher for professionalism (all work terms) Co-op Student Performance Evaluation (CSPE) (Supplementary Figure S3): Overall rating of “marginal” or higher (all work terms)
Midpoint assessment	Modified Angoff process used to set passing score (generally approximately 60%)
Annual OSCE	Modified Angoff process used to set passing score (generally approximately 60%)
Direct patient care rotations *	Overall grade of 70% or higher comprised of: Patient care skills (85%) assessed using the Ontario Pharmacy Patient Care Assessment Tool (OPPCAT) at 2 weeks (formative), 4 weeks (25%), and 8 weeks (60%) Interprofessional practice (5%), where students’ inter-professional practice is evaluated by an evaluator from a different profession in a summative manner Community of practice assignment (10%), where students address practice gaps within the community by sharing new information with health professionals and/or providing a service to patients
PEBC Pharmacist Qualifying Examination	MCQ and OSCE scores

* To facilitate analysis, the following data modifications were made: Didactic courses: PHARM 126: Calculations course excluded as it was a credit/no credit course with extreme imbalance between credit (94%) and no credit (6%) outcomes. PharmD Inventory of Skills: Nine quantitative scores/competencies are assessed per student. Given variability in applicable competencies per work term, performance on each competency was averaged across the 3 work terms for each student. Co-op Student Performance Evaluation: Ratings were combined into the categories of good and below, very good, excellent, and outstanding, and assigned numerical scores of 1–4, respectively, due to the limited number of marginal and satisfactory ratings (*n* = 7) compared to the number of good, very good, excellent, and outstanding ratings.

**Table 2:** Statistically significant relationships for analysis 1.

Course	Estimated Coefficient β	*p*-Value
OPPCAT only as predictor variable for rotation performance with PEBC MCQ as dependent variable		
PHARM 111: Anatomy and Physiology 2	−3.46	*p* = 0.001
PHARM 229: Professional Practice 4	3.87	*p* = 0.03
PHARM 321: Integrated Patient-Focused Care 6	−3.17	*p* = 0.03
PHARM 324: Integrated Patient-Focused Care 8	3.95	*p* = 0.002
PHARM 330: Professional Practice 6	−2.89	*p* = 0.04
Year 4 OSCE score	1.55	*p* = 0.03
Midpoint MCQ score	2.50	*p* < 0.001
Rotation 3 OPPCAT score	1.74	*p* = 0.04
OPPCAT only as predictor variable for rotation performance with PEBC OSCE as dependent variable		
PHARM 111: Anatomy and Physiology 2	−2.98	*p* = 0.01
PHARM 228: Professional Practice 3	2.40	*p* = 0.04
PHARM 229: Professional Practice 4	3.72	*p* = 0.04
PHARM 350: Fundamentals of Business Administration and Management	−2.39	*p* = 0.05
PHARM 425: Symposium	1.93	*p* = 0.03
Rotation course overall as predictor variable for rotation performance with PEBC MCQ as dependent variable		
PHARM 111: Anatomy and Physiology 2	−3.43	*p* = 0.002
PHARM 229: Professional Practice 4	3.65	*p* = 0.04
PHARM 321: Integrated Patient-Focused Care 6	−3.15	*p* = 0.03
PHARM 324: Integrated Patient-Focused Care 8	3.97	*p* = 0.003
PHARM 330: Professional Practice 6	−2.70	*p* = 0.05
Year 4 OSCE score	1.54	*p* = 0.03
Midpoint MCQ score	2.52	*p* < 0.001
Rotation course overall as predictor variable for rotation performance with PEBC OSCE as dependent variable		
PHARM 111: Anatomy and Physiology 2	−2.98	*p* = 0.01
PHARM 228: Professional Practice 3	2.56	*p* = 0.03
PHARM 229: Professional Practice 4	3.69	*p* = 0.05
PHARM 350: Fundamentals of Business Administration and Management	−2.57	*p* = 0.04
PHARM 425: Symposium	1.93	*p* = 0.03

**Table 3:** Statistically significant relationships for analysis 2.

Course	Estimated Coefficient β	*p*-Value
OPPCAT only from first completed rotation as dependent variable		
PHARM 222: Integrated Patient-Focused Care 3	−0.29	*p* = 0.02
PHARM 232: Medical Microbiology	−0.20	*p* = 0.02
PHARM 422: Integrated Patient-Focused Care 9	0.32	*p* < 0.001
OPPCAT only from second completed rotation as dependent variable		
Graduation year 2017	3.21	*p* = 0.04
PHARM 124: Pharmaceutics 1	0.26	*p* = 0.01
PHARM 220: Integrated Patient-Focused Care 1	0.28	*p* = 0.02
PHARM 222: Integrated Patient-Focused Care 3	−0.24	*p* = 0.03
PHARM 227: Health Systems in Society	−0.23	*p* < 0.001
PHARM 228: Professional Practice 3	0.33	*p* < 0.001
OPPCAT only from third completed rotation as dependent variable		
PHARM 129: Professional Practice 1	−0.20	*p* = 0.04
PHARM 222: Integrated Patient-Focused Care 3	−0.40	*p* < 0.001
PHARM 350: Fundamentals of Business Administration & Management	0.24	*p* = 0.03
PHARM 422: Integrated Patient-Focused Care 9	0.28	*p* < 0.001
CSPE overall (work term 1)	0.84	*p* = 0.03
Co-op direct patient care scores	1.95	*p* = 0.04
PHARM 430 (Primary Care) rotation overall score as dependent variable		
Graduation year 2018	−2.44	*p* = 0.03
PHARM 155: Drug Information Fundamentals	−0.19	*p* = 0.02
PHARM 321: Integrated Patient-Focused Care 6	0.27	*p* = 0.03
PHARM 422: Integrated Patient-Focused Care 9	0.22	*p* < 0.001
PHARM 440 (Primary Care) rotation overall score as dependent variable		
Graduation year 2017	2.23	*p* = 0.03
PHARM 124: Pharmaceutics 1	0.22	*p* = 0.01
PHARM 125: Pharmaceutics 2	−0.18	*p* = 0.04
PHARM 228: Professional Practice 3	0.17	*p* = 0.04
PHARM 232: Medical Microbiology	−0.17	*p* = 0.01
PHARM 422: Integrated Patient-Focused Care 9	0.17	*p* = 0.01
PHARM 450 (Elective) rotation overall score as dependent variable		
PHARM 129: Professional Practice 1	−0.21	*p* = 0.01
PHARM 222: Integrated Patient-Focused Care 3	−0.38	*p* < 0.001
PHARM 228: Professional Practice 3	0.22	*p* = 0.01
PHARM 252: Institutional Pharmacy Practice	0.16	*p* = 0.04
PHARM 320: Integrated Patient-Focused Care 5	0.26	*p* = 0.01
PHARM 422: Integrated Patient-Focused Care 9	0.22	*p* < 0.001
PHARM 425: Symposium	0.15	*p* = 0.02
Year 1 OSCE score	−0.10	*p* = 0.04
CSPE overall (work term 1)	0.66	*p* = 0.04
Co-op direct patient care scores	2.07	*p* = 0.01

**Table 4:** Course, OSCE, midpoint exam, OPPCAT and PEBC failures, and near failures by co-op performance.

	Overall Co-Op Rating below Good across One or More Work Terms (n = 7)	Overall Co-Op Rating of Good across One or More Work Terms (n = 44)	Overall Co-Op Rating of Good or above across All Work Terms (n = 336)	Overall Co-Op Rating of Very Good or above across All Work Terms (n = 292)
Performance in PharmD Program (n, %)				
Failure of at least one course	1 (14%)	4 (9%)	14 (4%)	11 (4%)
Near failure of at least one course	5 (71%)	31 (71%)	183 (55%)	157 (54%)
Failure of at least one annual OSCE	5 (71%)	30 (68%)	165 (49%)	140 (48%)
Near failure of at least one annual OSCE	5 (71%)	34 (77%)	248 (74%)	219 (75%)
Failure of midpoint assessment	2 (29%)	7 (16%)	46 (14%)	41 (14%)
Near failure of midpoint assessment	3 (43%)	25 (57%)	117 (35%)	95 (33%)
Failure of at least one OPPCAT	0 (0%)	0 (0%)	5 (2%)	5 (2%)
Near failure of at least one OPPCAT	4 (57%)	10 (23%)	31 (9%)	25 (9%)
Performance on PEBC Pharmacist Qualifying Examination (n, %)				
Failure of Part I (MCQ)	3 (43%)	6 (14%) *	24 (7%) ^±^	21 (7%) ^Δ^
Failure of Part II (OSCE)	1 (14%)	6 (14%) *	11 (3%) ^±^	6 (2%) ^Δ^

* n = 43 due to missing data; ^±^ n = 327 due to missing data; ^Δ^ n = 284 due to missing data.

## Data Availability

No new data were created or analyzed in this study. Data sharing is not applicable to this article.

## Supplementary Materials

The following supporting information can be downloaded at: https://www.mdpi.com/article/10.3390/pharmacy12030090/s1. The following supporting information has been submitted as a separate document, Figure S1: UW PharmD curricular grid; Table S1: abbreviations; Figure S2: PharmD inventory of skills evaluation, Table S2: high-correlation coefficients between predictor variables; Figure S3: Co-op Student Performance Evaluation.
