# Supplementary material for: The Association between Academic Performance and Entry-to-Practice Milestones within a Co-Operative Education PharmD Program
Source: Pharmacy (Basel). 2024 Jun 11;12(3):90. doi: 10.3390/pharmacy12030090 (PMC11207931; doi:10.3390/pharmacy12030090)
Supplement: Supplementary file 1 [file pharmacy-12-00090-s001.zip › pharmacy-3015697-supplementary.pdf]

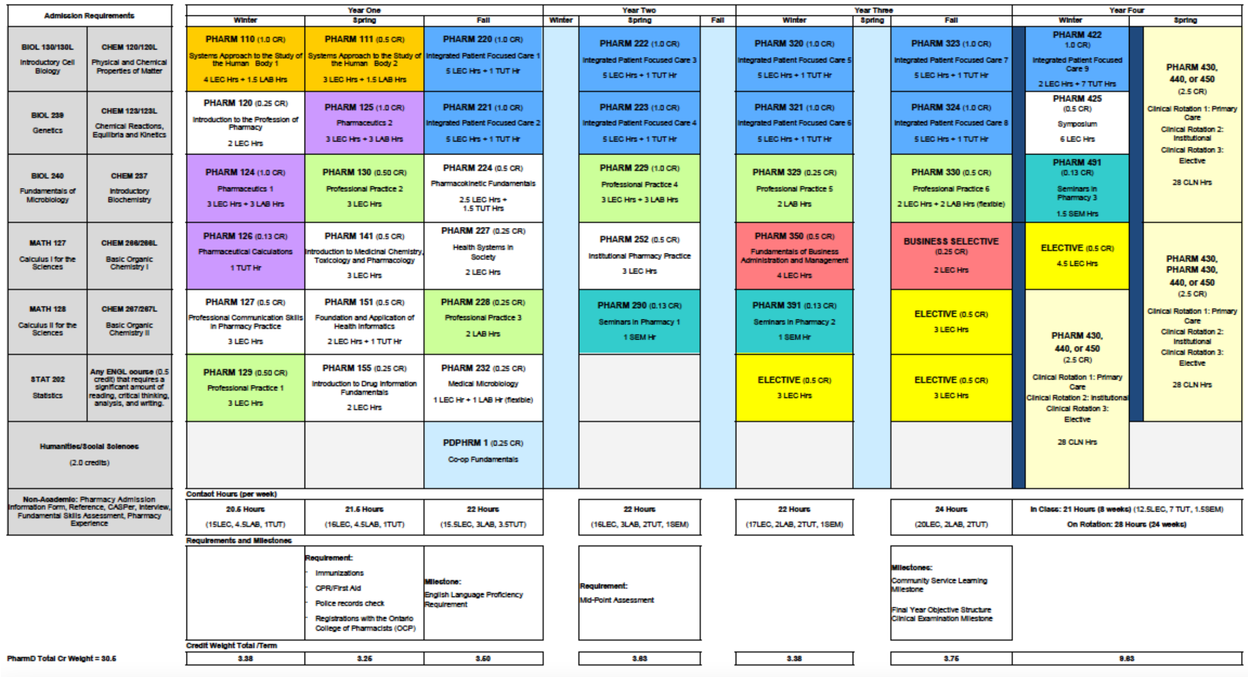

Figure S1: <institution blinded> Curricular Grid

Table S1: Abbreviations

Abbreviation	Explanation
APPE	Advanced Pharmacy Practice Experience
Co-op	Cooperative Education
CSL	Community Service Learning
GPA	Grade Point Average
IPFC	Integrated Patient Focused Care
MCQ	Multiple-Choice Question
NAPLEX	North American Pharmacist Licensure Exam
OPPCAT	Ontario Pharmacy Patient Care Assessment
OSCE	Objective Structured Clinical Examination
PCAT	Pharmacy College Admissions Test
PEBC	Pharmacy Examining Board of Canada
PharmD	Doctor of Pharmacy
PP	Professional Practice
QE	Qualifying Exam
<institution blinded>	<institution blinded>
WIL	Work Integrated Learning

Institution Blinded

Student:
Preceptor:
Site:

Figure S2: PharmD Inventory of Skills Evaluation

Date: ()

YOU ARE CURRENTLY IN PREVIEW MODE - THE EVALUATION CANNOT BE SUBMITTED.

Start Evaluation

PharmD Co-Op Inventory of Skills

How to apply scoring

Within the **1st or 2nd co-op term**, the student must have achieved at least an **overall** **"Good" in Distribution competencies.** As some competencies may not be achieved in every setting, a **maximum of 2 "not applicable" are acceptable.** Please note that if the work term does not contain a dispensing component, the **Distribution** sections should not be completed.

Within the **2nd or 3rd co-op term,** student must have achieved at least an **overall** **"Good" in Patient Care competencies.** As some competencies may not be achieved in every setting, **a maximum of 2 "not applicable" are acceptable.** Please note that if the work term does not contain a patient care component, the **Direct Patient Care** sections should not be completed.

For **all co-op terms**, student must have achieved at least an **overall** **"Good"** in Communication competencies, and must also **overall** **"Meet expectations"** in Professionalism competencies.

To accurately access the student, please observe them at least 5 times performing the activity. If it is a discussion activity, it is only required that the student has a clear understanding of the process or documentation.

Communication Competencies	MIDPOINT	FINAL
**Demonstrates written communication skills, allowing the reader to logically develop a clear idea of the intended**	○ Superior<br>○ Excellent<br>○ Good<br>○ Developing<br>Unsatisfactory	*Required*<br>○ Superior<br>○ Excellent<br>○ Good<br>○ Developing

**meaning of the note or document.**	○	○ Unsatisfactory
**Selects communication language which is appropriate for the target audience - e.g. patients, other health care professionals.**	○ Superior<br>○ Excellent<br>○ Good<br>○ Developing<br>○ Unsatisfactory	***Required***<br>○ Superior<br>○ Excellent<br>○ Good<br>○ Developing<br>○ Unsatisfactory
**When speaking, uses organized processes; uses listening skills; uses verbal techniques to maximize understanding (e.g. repeating back verbal orders, using recognized terminology).**	○ Superior<br>○ Excellent<br>○ Good<br>○ Developing<br>○ Unsatisfactory	***Required***<br>○ Superior<br>○ Excellent<br>○ Good<br>○ Developing<br>○ Unsatisfactory

Communication Overall Rating

	MIDPOINT	FINAL
**Overall Rating for Communication**	○ Superior<br>○ Excellent<br>○ Good<br>○ Developing<br>○ Unsatisfactory	***Required***<br>○ Superior<br>○ Excellent<br>○ Good<br>○ Developing<br>○ Unsatisfactory

Communication - comments are mandatory if a rating of “unsatisfactory” or “developing” is given

Question Comments (Midpoint):	Question Comments (Final):
Enter Midpoint comments	Enter Final comments

Distribution	MIDPOINT	FINAL
**Please note that if the co-op position does not contain a distribution component, questions may be left blank in this section.**		
**Addresses concerns related to the validity, clarity, completeness or authenticity of the prescription adhering to applicable regulations and legislation.**	Superior<br>Excellent<br>Good<br>Developing<br>Unsatisfactory	Superior<br>Excellent<br>Good<br>Developing<br>Unsatisfactory
**Reviews prescriptions for dosage, frequency, appropriateness of therapy. Monitors allergies, intolerances and/or adverse reactions. Reviews any interactions and discusses with supervisor.**	Superior<br>Excellent<br>Good<br>Developing<br>Unsatisfactory	Superior<br>Excellent<br>Good<br>Developing<br>Unsatisfactory
**Clarifies missing prescription information, or to obtain further patient information (with physician, nurse, chart or other appropriate means).**	Superior<br>Excellent<br>Good<br>Developing<br>Unsatisfactory	Superior<br>Excellent<br>Good<br>Developing<br>Unsatisfactory

Distribution	MIDPOINT	FINAL
**Please note that if the co-op position does not contain a distribution component, questions may be left blank in this section.**		

As some competencies may not be acheived in every setting, a **maximum of 2 "not applicables" are acceptable where possible in this section.**

	Midpoint	Final
**Checks the final product and labelling using systematic approach, including independent double check (e.g. by co-op supervisor). Properly utilizes auxiliary labels and patient instruction aids as appropriate.**	○ Not Applicable<br>○ Superior<br>○ Excellent<br>○ Good<br>○ Developing<br>○ Unsatisfactory	○ Not Applicable<br>○ Superior<br>○ Excellent<br>○ Good<br>○ Developing<br>○ Unsatisfactory
**Prepares and compounds products using appropriate labelling and expiry dates.**	○ Not Applicable<br>○ Superior<br>○ Excellent<br>○ Good<br>○ Developing<br>○ Unsatisfactory	○ Not Applicable<br>○ Superior<br>○ Excellent<br>○ Good<br>○ Developing<br>○ Unsatisfactory
**Knowledge of inventory management (e.g., cold packaging, ordering, recalls, expired products, safeguarding of controlled substances). If the student is not directly involved, can be a discussion.**	○ Not Applicable<br>○ Superior<br>○ Excellent<br>○ Good<br>○ Developing<br>○ Unsatisfactory	○ Not Applicable<br>○ Superior<br>○ Excellent<br>○ Good<br>○ Developing<br>○ Unsatisfactory
**Understands financial aspects of pharmacy; billing processes, 3rd party payment, ODB codes in community pharmacy or formulary management in hospital pharmacy. If the student is not directly involved, can be a discussion.**	○ Not Applicable<br>○ Superior<br>○ Excellent<br>○ Good<br>○ Developing<br>○ Unsatisfactory	○ Not Applicable<br>○ Superior<br>○ Excellent<br>○ Good<br>○ Developing<br>○ Unsatisfactory

**Distribution Overall Rating**	**MIDPOINT**	**FINAL**

Please note that if the co-op position does not contain a distribution component, questions may be left blank in this section.

Please provide a "general" or "global" overall rating for the student's distribution abilities.

**Overall Rating**<br><br>Distribution related - comments are mandatory if a rating of “unsatisfactory” or “developing” is given	○ Superior<br>○ Excellent<br>○ Good<br>○ Developing<br>○ Unsatisfactory	○ Superior<br>○ Excellent<br>○ Good<br>○ Developing<br>○ Unsatisfactory

**Question Comments (Midpoint):**	**Question Comments (Final):**
Enter Midpoint comments	Enter Final comments

Direct Patient Care

Please note that in the 1st workterm (January-April), it is <u>NOT</u> required to complete this portion, other than for general feedback to the student. Questions may be left blank in this section.

	MIDPOINT	FINAL

**Demonstrates empathy and sensitivity when interacting with patients in order to meet their needs.**	○ Superior<br>○ Excellent<br>○ Good<br>○ Developing<br>○ Unsatisfactory	○ Superior<br>○ Excellent<br>○ Good<br>○ Developing<br>○ Unsatisfactory
**Utilizes interview techniques to ask appropriate questions, so as to delineate patient presentation (e.g. SCHOLAR: Symptoms, Characteristics, History, Onset, Location, Aggravating factors, Remitting factors or other information gathering techniques.)**	○ Superior<br>○ Excellent<br>○ Good<br>○ Developing<br>○ Unsatisfactory	○ Superior<br>○ Excellent<br>○ Good<br>○ Developing<br>○ Unsatisfactory
**Can provide patient education/counseling on common drugs for new prescriptions. Has ability to perform foundational level of educating patient on proper dose, frequency, method of administration, duration of usage, expected outcomes and storage of medication.**	○ Superior<br>○ Excellent<br>○ Good<br>○ Developing<br>○ Unsatisfactory	○ Superior<br>○ Excellent<br>○ Good<br>○ Developing<br>○ Unsatisfactory
**Using typical pharmacy resources, responds appropriately to common questions (related to top commonly dispensed drugs). Utilizes available drug info resources.**	○ Superior<br>○ Excellent<br>○ Good<br>○ Developing<br>○ Unsatisfactory	○ Superior<br>○ Excellent<br>○ Good<br>○ Developing<br>○ Unsatisfactory

**Direct Patient Care**	**MIDPOINT**	**FINAL**
**Please note that in the 1st workterm, it is <u>NOT</u> required to complete this, other than**		

for feedback for the student and questions can be left blank.

**Is able to identify basic, simple drug-related problems with commonly dispensed drugs and common diseases.**	○ Not Applicable<br>○ Superior<br>○ Excellent<br>○ Good<br>○ Developing<br>○ Unsatisfactory	○ Not Applicable<br>○ Superior<br>○ Excellent<br>○ Good<br>○ Developing<br>○ Unsatisfactory
**Can assess possible treatment options using an evidence-based approach outlining benefit and risks.**	○ Not Applicable<br>○ Superior<br>○ Excellent<br>○ Good<br>○ Developing<br>○ Unsatisfactory	○ Not Applicable<br>○ Superior<br>○ Excellent<br>○ Good<br>○ Developing<br>○ Unsatisfactory
**Monitors outcomes (e.g. calling the patient to follow up on a new prescription or drug-related problem), and adjusts therapy as appropriate.**	○ Not Applicable<br>○ Superior<br>○ Excellent<br>○ Good<br>○ Developing<br>○ Unsatisfactory	○ Not Applicable<br>○ Superior<br>○ Excellent<br>○ Good<br>○ Developing<br>○ Unsatisfactory
**Can provide patient education/counseling on OTC (over-the-counter) products. Has ability to perform foundational level of educating patient on proper dose, frequency, method of administration, duration of usage, expected outcomes and storage of product. Can determine appropriateness for self-care, and refers patient to other health care professionals when suitable.**	○ Not Applicable<br>○ Superior<br>○ Excellent<br>○ Good<br>○ Developing<br>○ Unsatisfactory	○ Not Applicable<br>○ Superior<br>○ Excellent<br>○ Good<br>○ Developing<br>○ Unsatisfactory
	○ Not Applicable	○ Not Applicable

	Midpoint	Final
**Can demonstrate the foundational level of the Ontario expanded scope of practice - e.g. administer a substance by injection or inhalation for the purpose of education and demonstration; prescribe specified drug products for smoking cessation only.**	○ Superior<br>○ Excellent<br>○ Good<br>○ Developing<br>○ Unsatisfactory	○ Superior<br>○ Excellent<br>○ Good<br>○ Developing<br>○ Unsatisfactory
**When within Ontario, has the ability to perform the foundational level of renewing & adapting prescriptions (alter dose, dosage form, regimen, or route of administration), while keeping the prescriber informed.**		

Direct Patient Care Overall Rating

Please provide a "general" or "global" overall rating for the student's patient care abilities.

	MIDPOINT	FINAL
**Overall Rating**	○ Superior<br>○ Excellent<br>○ Good<br>○ Developing<br>○ Unsatisfactory	○ Superior<br>○ Excellent<br>○ Good<br>○ Developing<br>○ Unsatisfactory

Direct Patient Care - comments are mandatory if a rating of “unsatisfactory” or “developing” is given.

Question Comments (Midpoint):	Question Comments (Final):
Enter Midpoint comments	Enter Final comments

Professionalism	MIDPOINT	FINAL
**Accepts responsibility and accountability for own actions and decisions.**	○ Meets expectations<br>○ Does NOT meet expectations	***Required***<br>○ Meets expectations<br>○ Does NOT meet expectations
**Demonstrates respect for privacy and confidentiality of the patient.**	○ Meets expectations<br>○ Does NOT meet expectations	***Required***<br>○ Meets expectations<br>○ Does NOT meet expectations
**Acknowledges, accepts and applies constructive feedback to identify limitations or strengths.**	○ Meets expectations<br>○ Does NOT meet expectations	***Required***<br>○ Meets expectations<br>○ Does NOT meet expectations
**Utilizes time efficiently, is prepared for patient encounters and demonstrates reliability, dependability and punctuality.**	○ Meets expectations<br>○ Does NOT meet expectations	***Required***<br>○ Meets expectations<br>○ Does NOT meet expectations
**Maintains professional appearance, and follows site policies & procedures.**	○ Meets expectations<br>○ Does NOT meet expectations	***Required***<br>○ Meets expectations<br>○ Does NOT meet expectations
**Is respectful and cooperative with colleagues and others, and respects patient & families' rights.**	○ Meets expectations<br>○ Does NOT meet expectations	***Required***<br>○ Meets expectations<br>○ Does NOT meet expectations

Professionalism Overall Rating	MIDPOINT	FINAL
**Overall Rating**	○ Meets expectations<br>○ Does NOT meet expectations	***Required***<br>○ Meets expectations<br>○ Does NOT meet expectations

Professionalism - comments are mandatory if a rating of "does not meet expectations" is given

Question Comments (Midpoint):	Question Comments (Final):
Enter Midpoint comments	Enter Final comments

**Student:**	**MIDPOINT**	**FINAL**
**I have discussed this Inventory of Skills evaluation with my student.**	○ Yes<br>○ No	***Required***<br>○ Yes<br>○ No

Student: Comments (Midpoint):	Student: Comments (Final):
Enter Midpoint comments	Enter Final comments

Back to Top

Adobe PDF Reader is required to save and submit this form.

Save As | E-mail Form

Figure S3: Co-op Student Performance Evaluation

Institution Blinded **CO-OPERATIVE EDUCATION** Institution Blinded

Student Performance Evaluation

Student Name ______________________ **Organization** ______________________

Student ID Number ______________________ **Student Job Title** ______________________

Term ☐ **Winter (Jan-Apr)** ☐ **Spring (May-Aug)** ☐ **Fall (Sept – Dec)** **Year: 20____**

Supervisor's Guidelines for Completion

MID-TERM REVIEW (Informal)

Please conduct a mid-term review with your student to assist in their progress during the work term. Using this form as a guideline, the mid-point discussion is an opportunity for the supervisor and student to discuss topics such as:

- Progress towards overall expectations and goals
- Student's work performance so far
- Training or mentoring resources required for remainder of work term

END OF TERM EVALUATION (Required)

The end-of-term performance evaluation allows the supervisor and student to fulfill the evaluation process.

The return of this completed evaluation form is required for the student to receive credit for the work term.

Please fill out this form near the end of the student's work term.

Guidance on the Rating Scale

Performance Expectations -these scales measure the behaviours and abilities that all co-op students are expected to progressively attain and refine as they advance through their years of study

Developing Performance (1-2)
Students performing within this range require further development and support to meet the performance expectations with respect to output, quality standards, delivery of goals and/or assignments.

Good Performance (3-5)
Students performing within this range are meeting and, in some instances, exceeding the performance expectations in respect to output, quality standards, and delivery of goals and/or assignments.

Superior Performance (6-7)
Students performing within this range are consistently exceeding the performance expectations and they should be demonstrating the ability to take on broader responsibilities that would normally be reserved for a staff member working in a regular/permanent role (non-coop).

Problem Solving. *Ability to analyze problems or procedures, evaluate alternatives, and select best course of action.*

1	2	3	4	5	6	7	Not observed
Developing Performance		*Good Performance*			*Superior Performance*		
• Can make routine decisions but needs ongoing guidance and checking		• Can be relied upon to make good decisions, requires limited guidance			• Able to manage complex decisions for work without guidance		

EXAMPLE

1. **Interest in Work.** *The degree to which the student pursues goals with commitment and takes pride in accomplishments.*		
☐ 1 ☐ 2	☐ 3 ☐ 4 ☐ 5	☐ 6 ☐ 7 ☐ **Not observed**
Developing Performance<br>• Shows little enthusiasm for assigned work, infrequently requests additional tasks	*Good Performance*<br>• Enthusiastic about their assignments/work, agreeable to new responsibilities, asks for new tasks	*Superior Performance*<br>• Displays enthusiasm for work that is beyond their job requirements; proactively seeks new tasks and responsibilities

2. **Ability to Learn**. *The extent to which the student becomes proficient with job duties and work processes.*		
☐ 1 ☐ 2	☐ 3 ☐ 4 ☐ 5	☐ 6 ☐ 7 ☐ **Not observed**
Developing Performance<br>• Sometimes slow to become proficient at new tasks or work processes	*Good Performance*<br>• Quick to become proficient at new tasks	*Superior Performance*<br>• Exceeds expectations in the complexity and difficulty of work they are able to successfully complete

3. **Quality of Work.** *The ability of the student to set high standards for own personal performance; strive for quality work; put forth extra effort to ensure quality work.*		
☐ 1 ☐ 2	☐ 3 ☐ 4 ☐ 5	☐ 6 ☐ 7 ☐ **Not observed**
Developing Performance<br>• Work does not meet expectations, has more than the expected number of errors	*Good Performance*<br>• Work is usually very thorough and well done, few errors	*Superior Performance*<br>• Work is always very thorough and of excellent quality, few if any errors

4. **Quantity of Work.** *The volume of work produced by the student, along with his or her speed and consistency of output.*		
☐ 1 ☐ 2	☐ 3 ☐ 4 ☐ 5	☐ 6 ☐ 7 ☐ **Not observed**
Developing Performance<br>• Does not always complete work within time limits	*Good Performance*<br>• Completes the majority of work within specified deadlines	*Superior Performance*<br>• Consistently completes work ahead of schedule; seeks additional tasks

5. **Problem Solving.** *The student's demonstrated ability to analyze problems or procedures, evaluate alternatives, and select the best course of action.*		
☐ 1 ☐ 2	☐ 3 ☐ 4 ☐ 5	☐ 6 ☐ 7 ☐ **Not observed**
Developing Performance<br>• Can make routine decisions but needs guidance and checking	*Good Performance*<br>• Can be relied upon to make good decisions, requires limited guidance	*Superior Performance*<br>• Independently manages complex tasks and makes good decisions for work without guidance

6. **Teamwork.** *The degree to which the student works well in a team setting.*		
☐ 1 ☐ 2	☐ 3 ☐ 4 ☐ 5	☐ 6 ☐ 7 ☐ **Not observed**
Developing Performance<br>• Sometimes uncooperative; or experiences difficulty relating to others	*Good Performance*<br>• Frequently cooperative, good team worker	*Superior Performance*<br>• Consistently cooperative, proactively seeks to improve working relationships

**7. Dependability.** *The manner in which the student conducts his or herself in the working environment.*		
☐ 1 ☐ 2	☐ 3 ☐ 4 ☐ 5	☐ 6 ☐ 7 ☐ **Not observed**
Developing Performance<br>• Displays an inconsistent work ethic and does not always report to work on time or has some attendance issues	*Good Performance*<br>• Displays a strong work ethic and is present at work and meetings in a reliable and timely manner	*Superior Performance*<br>• Displays an excellent work ethic and volunteers to adapt personal schedule to meet work demands

**8. Response to Supervision.** *The manner in which the student responds to direction and constructive criticism.*		
☐ 1 ☐ 2	☐ 3 ☐ 4 ☐ 5	☐ 6 ☐ 7 ☐ **Not observed**
Developing Performance<br>• Sometimes disregards direction and feedback from supervisor	*Good Performance*<br>• Integrates feedback from supervisor into their work to improve productivity & efficiency	*Superior Performance*<br>• Takes the initiative to follow through on all feedback from supervisor and to continuously improve upon their daily tasks and approach to work

**9. Reflection.** *The student's demonstrated ability to learn and adapt from previous experience.*		
☐ 1 ☐ 2	☐ 3 ☐ 4 ☐ 5	☐ 6 ☐ 7 ☐ **Not observed**
Developing Performance<br>• Has to be told many times before they modify their behaviour or approach to new work based on errors in previous performance.	*Good Performance*<br>• Occasionally needs reminder to modify their behaviour or approach to new work based on errors in previous performance	*Superior Performance*<br>• Independently recognizes the errors in previous performance and proactively modifies their behaviour and approach to new work

**10. Resourcefulness**. *The student's demonstrated ability to develop innovative solutions and display flexibility in unique or demanding circumstances.*		
☐ 1 ☐ 2	☐ 3 ☐ 4 ☐ 5	☐ 6 ☐ 7 ☐ **Not observed**
Developing Performance<br>• Unsure how to approach new or stressful situations; has difficulty adjusting to changing priorities and circumstances	*Good Performance*<br>• Responds appropriately to new or stressful situations; can adjust to changing priorities and circumstances with guidance	*Superior Performance*<br>• Generates effective resolutions to new or stressful situations; readily adjusts to changing priorities and circumstances

**11. Ethical Behaviour**. *The extent to which the students behaviour demonstrates integrity and ethics in work and relationships.*		
☐ 1 ☐ 2	☐ 3 ☐ 4 ☐ 5	☐ 6 ☐ 7 ☐ **Not observed**
Developing Performance<br>• Needs guidance in making appropriate choices to avoid questionable conduct and/or a conflict of personal and professional interests	*Good Performance*<br>• Is able to make the appropriate choices to avoid questionable conduct and/or a conflict of personal and professional interests	*Superior Performance*<br>• Proactively identifies potential conflicts of interest or questionable conduct and acts to avoid or mitigate these issues

**12. Appreciation of Diversity.** *The degree to which the student shows understanding and sensitivity to needs and differences of others (i.e. ethnicity, religion, language, etc.)*			
☐ **1** ☐ **2**	☐ **3** ☐ **4** ☐ **5**	☐ **6** ☐ **7**	☐ **Not observed**
Developing Performance<br>• Has difficultly interacting with others due to individual differences	*Good Performance*<br>• Has positive interactions with others and is respectful of individual differences	*Superior Performance*<br>• Demonstrates leadership in promoting positive interactions and encouraging others to work together despite individual differences	

**13. Entrepreneurial Orientation.** *The students demonstrated ability to take informed risks that demonstrate creativity and add value to the company.*			
☐ **1** ☐ **2**	☐ **3** ☐ **4** ☐ **5**	☐ **6** ☐ **7**	☐ **Not observed**
Developing Performance<br>• Has difficulty evaluating alternative ideas and making choices that enhance the department or organization	*Good Performance*<br>• Able to evaluate alternative ideas and will sometimes make choices that that enhance the department or organization	*Superior Performance*<br>• Able to effectively evaluate alternative ideas and independently makes choices that enhance the department or organization	

**14. Written Communication.** *The extent to which the student demonstrates effective written communication.*			
☐ **1** ☐ **2**	☐ **3** ☐ **4** ☐ **5**	☐ **6** ☐ **7**	☐ **Not observed**
Developing Performance<br>• Not consistently clear and concise or requires frequent checking and editing	*Good Performance*<br>• Normally clear, well organized and understandable and needs only moderate checking and editing	*Superior Performance*<br>• Always clear, well organized and easily understandable; rarely requires checking and editing	

**15. Oral Communication**. *The extent to which the student demonstrates effective oral communication.*			
☐ **1** ☐ **2**	☐ **3** ☐ **4** ☐ **5**	☐ **6** ☐ **7**	☐ **Not observed**
Developing Performance<br>• Occasionally encounters difficulty with expressing ideas clearly and persuasively; demonstrates discomfort with public speaking	*Good Performance*<br>• Normally clear, well organized, understandable, and persuasive, good public speaker	*Superior Performance*<br>• Always clear, well organized, easily understandable, and exceptionally persuasive, excellent public speaker	

**16. Interpersonal Communication.** *The extent to which the student effectively listens, conveys, and receives ideas, information, and direction.*			
☐ **1** ☐ **2**	☐ **3** ☐ **4** ☐ **5**	☐ **6** ☐ **7**	☐ **Not observed**
Developing Performance<br>• Displays inconsistent listening skills and is reluctant to seek input from others.	*Good Performance*<br>• Interactions with others demonstrate acceptable listening skills and the ability to sometimes seek the opinions, ideas, and expertise of others.	*Superior Performance*<br>• Interactions with others demonstrate exceptional active listening skills and the ability to proactively seek the opinions, ideas, and expertise of others.	

Overall Performance Rating

Rating	
**Outstanding Performance**<br>• The student has significantly exceeded all behavioural and developmental performance expectations in respect to output, quality standards, delivery of goals and assignments.<br>• This rating is **reserved for only those few students** who have distinguished themselves by their unique contribution or exceptional performance<br><br>**Your written comments are required below in order to register the rating of Outstanding**	☐
**Excellent Performance**<br>• The student has exceeded all performance expectations in respect to output, quality standards, delivery of goals and assignments.<br>• Receiving this rating means the manager is delighted with this student's performance.	☐
**Very Good Performance**<br>• The student has met all and exceeded some performance expectations in respect to output, quality standards, delivery of goals and assignments.<br>• Receiving this rating means the manager is very pleased with this student's performance.	☐
**Good Performance**<br>• The student meets performance expectations in respect to output, quality standards, delivery of goals and assignments.<br>• Receiving this rating means the manager is pleased with this student's performance.	☐
**Satisfactory Performance**<br>• The student has not fully met the performance expectations in respect to output, quality standards, delivery of goals and assignments<br>• Receiving this rating means the manager is mostly satisfied with the student's performance	☐
**Marginal Performance**<br>• Overall performance requires improvement and/or certain key aspects of performance require improvement while other aspects may be satisfactory<br>• Receiving this rating means the manager is displeased with this student's performance	☐
**Unsatisfactory Performance**<br>• The student did not meet performance requirements.	☐

Supervisor's Comments - Please comment on the student's overall job performance:

Student's Comments - Please comment on your overall performance including your ability to achieve learning objectives and your future employment expectations:

Supervisor's Recommendations- Please provide your recommendations for the student's personal and/or professional development (optional):

*** Did you review the completed evaluation form with the student? (Please ensure the student has a copy)**

Yes ☐ No ☐

Next Work Term

Do you wish to have the student return for the next work term?

Yes ☐ No ☐ Not Applicable ☐

If yes, have you offered to re-employ the student for the next work term?

Yes ☐ No ☐ To be determined ☐

If yes, was your offer: Accepted ☐ Declined ☐

If the student, has accepted please confirm:

Work term Dates: From: _________ To: __________ To be determined ☐

Co-operative Education will contact you to confirm new job details.

____

Supervisor's Name (Please Print) **Signature** **Title** **Date**

____

Student's Signature **Date**

____

Manager/Human Resources Signature (optional) **Title** **Date**

Table S2: High correlation coefficients between predictor variables

**Pharm 111 (Anatomy and Physiology 2)**	**Pharm 221 (Integrated Patient Focused Care 2)**	**Pharm 320 (Integrated Patient Focused Care 5)**	**Pharm 321 (Integrated Patient Focused Care 6)**	**Pharm 228 (Professional Practice 3)**	**Pharm 329 (Professional Practice 5)**	**Pharm 450 (Clinical Rotation 3)**
Pharm 110 (Anatomy and Physiology 1); r=.81	Pharm 220 (Integrated Patient Focused Care 1); r=.73	Pharm 223 (Integrated Patient Focused Care 4); r=.73	Pharm 223 (Integrated Patient Focused Care 4); r=.72	Year 1 OSCE Score; r=.78	Year 3 OSCE Score; r=.8	Rotation 3 OPPCAT; r=.71
Pharm 125 (Pharmaceutics 2); r=.71		Pharm 321 (Integrated Patient Focused Care 6); r=.76	Pharm 320 (Integrated Patient Focused Care 5); r=.76			
Pharm 141 (Medicinal Chemistry, Toxicology and Pharmacology; r=.72		Pharm 323 (Integrated Patient Focused Care 7); r=.73				
